# Supplementary material for: RAS-pathway mutation patterns define epigenetic subclasses in juvenile myelomonocytic leukemia
Source: Nat Commun. 2017 Dec 19;8:2126. doi: 10.1038/s41467-017-02177-w (PMC5736667; doi:10.1038/s41467-017-02177-w)
Supplement: Supplementary file 1 — Supplementary Information [file 41467_2017_2177_MOESM1_ESM.docx]

**Supplementary Information**

**SUPPLEMENTARY FIGURE 1 Identification of JMML-specific aberrant DNA methylation patterns**

**a** Consensus clustering of the 5000 most variable unfiltered CpGs. Depicted are the 1000 most variable CpG probes. Samples (columns) are ordered according to consensus clustering results and clustered using Manhattan distance metric and Ward’s linkage. Somatic mutations in *PTPN11*, *NF1*, *KRAS*, *NRAS* or their absence (quadneg), diagnosis of Noonan syndrome, karyotype (presence of monosomy 7 [Mo7]) and relapse events (red) are annotated on top of the heatmap. The bottom annotation depicts the consensus cluster results based on unfiltered CpGs and on jmmlDMPs, respectively. One patient had missing clinical outcome data (grey).

**b-e** Two-dimensional principal component analysis (PCA) depicting the first 5 principal components (PC) of methylation dynamics across 12 normal hematopoietic cell types as described in **Figure 1a**. Colors encode the cell types. Each colored dot represents a single sample of a given cell type.

**f** Consensus clustering for the 5000 most variable nvCpGs using three groups (k=3). The consensus matrix shows pairwise cluster assignment frequencies derived from 500 iterations based on Manhattan distance metric and Ward’s linkage. Consensus values range from 0 (white) to 1 (dark blue). Patient samples with a high consensus value (1) always cluster together whereas samples with a low consensus value never cluster together.

**g, h** The consensus cumulative distribution function (CDF) plot **(f)** shows the cumulative distribution functions of the consensus matrix for each k. The Delta Area plot **(g)** displays the relative increase in the area under the curve (AUC) of the CDF plot. Together with the CDF plot, this plot allows to determine the cluster number (k) for which no relevant increase in AUC can be observed.

**i Boxplots depicting the distribution of** mean methylation levels per sample across all CpGs that are not dynamic during hematopoiesis (nvCpGs, n=308,199). Percentages of methylation are depicted for each methylation group (HM: red, LM: green). Boxes represent the interquartile range and whiskers depict the minimum and maximum of the distribution. P-values are calculated using the two-sided unpaired Welch’s t-test.

**SUPPLEMENTARY Figure 2 JMML-specific aberrant methylation patterns characterize three distinct JMML subgroups in a validation cohort**

Consensus clustering of the 1000 most variable jmmlDMPs in the validation cohort (n=147).

**a-d** Consensus cumulative distribution function (CDF) plot (**a**) and Delta Area plot (**b**) for consensus clustering of the validation cohort using the 1000 most variable jmmlDMPs. These plots clearly indicate that three clusters would be optimal to separate the samples from the validation cohort based on the JMML-specific methylation events. The consensus matrix (**c**) for the samples from the validation cohort (n=147) testing three clusters (k=3) shows the pairwise cluster assignment frequencies derived from 500 iterations based on Manhattan distance metric and Ward’s linkage. Patient samples with a high consensus value (1) always cluster together, whereas samples with a low consensus value (0) never cluster together. The item consensus (IC) plot (**d**) shows the IC values for each patient sample (item; vertical bars). The IC is the mean consensus value between an item and members of a consensus cluster and is depicted for two (k=2), three (k=3) and four (k=4) clusters. The colored asterisks on top represent the consensus clusters to which each sample is assigned (HM=red, IM=blue, and LM=green).

**SUPPLEMENTARY FIGURE 3 DNA methylation defines an aggressive JMML subgroup with high risk of relapse**

Kaplan-Meier curves showing the clinical outcome of JMML patients stratified for methylation subgroups. HM: red curve, IM: blue curve and LM: green curve. At the bottom of each graph the numbers of individuals at risk (N) and the numbers of events (E) are summarized according to methylation group. The curve labels represent the estimates for the 5-year time point and the 95% confidence interval of the estimate. The p-values were computed using log-rank test or Gray’s test as indicated.

**a-c** Overall survival from diagnosis (**a**), Event-free survival (**b**), and treatment-related mortality (TRM; **c**) from HSCT for all patients from the validation cohort with complete mutation analysis who received HSCT (n=92) and who did not have a diagnosis of Noonan-syndrome or CBL-syndrome (please refer to **Supplementary Tables 2 & 3 and to Supplementary Data 3** for further information on patient characteristics).

**d** Cumulative incidence of relapse (CIR) for patients with HLA-identical sibling donor or ≥9/10 HLA allele-level matched unrelated donor, uniform preparative regimen and immunosuppressive therapy according to EWOG study recommendations; n=47).

**SUPPLEMENTARY Figure 4 RAS-pathway mutation patterns and their association with JMML methylation subgroups**

**a, b** “Genotype”-specific DMPs were called for Noonan-syndrome patients and for *CBL*-mutated cases. Unsupervised clustering of Noonan-specific (**a**) and *CBL*-specific (**b**) DMPs in all patients from the validation cohort (n=147). Annotation for “genotype” (somatic mutations in *PTPN11*, *KRAS*, and *NRAS*, germline or somatic *CBL* mutations; clinical diagnosis of neurofibromatosis: NF1; quintuple-negative: quint.-neg.; Noonan: clinical diagnosis of Noonan-syndrome) and karyotype is depicted on top of the heatmap. DNA methylation levels are shown from light blue (0) to red (1).

**SUPPLEMENTARY Figure 5 Aberrant DNA methylation patterns are associated with signaling pathway activation and over-expression of DNMTs**

**a, b** Annotation of jmmlDMPs (n=5380) to non-coding regions and repetitive elements (**a**) and distribution of all nvCpGs (**b**), serving as the “background” distribution.

**c** Bubble plot showing the enrichment of histone marks in jmmlDMPs across 13 cell lines. Enrichment (red) and depletion (green) is shown for each histone mark for each cell line. The dot colors represent the logarithmic fold change and the dot size indicates the log(p)-value for each enrichment. The outline colors indicate statistical significance (black: significant, grey: not significant). Absent dots indicate missing data.

**d** Results of gene set enrichment analysis using the molecular signature database (MSigDB). The bar plots depict the top ten gene sets enriched across all jmmlDMPs based on p-values from the hypergeometric distribution ([http://software.broadinstitute.org/gsea/ msigdb/index.jsp](http://software.broadinstitute.org/gsea/%20msigdb/index.jsp)) {Subramanian, 2005 #963}.

**e** Oncoprint for exome sequencing data from JMML samples (n=50). One hundred forty-seven (147) genes known to be mutated in JMML were investigated for the presence of mutations (SNVs, indels, CNVs). Depicted are all genes with at least one mutation call in this sample set. The sum of genomic alterations (single nucleotide variants: snv [brown], copy number variations: cnv [green], insertions/deletions: indels [lilac]) is shown per sample at the top and per gene across all samples on the right. Annotation for methylation clusters is shown at the bottom.

**f** Expression of RAS-signaling genes (*KRAS*, *CBL*, *NRAS*, *NF1*, *RASFF1*) and genes involved in epigenetic regulation (*DNMT3A*, *TET1*, *EZH2*, *SUZ12*, *EED*). Depicted are quantile normalized gene expression microarray data from 15 JMML patients from the discovery cohort for whom RNA of sufficient quality was available. For the purpose of this analysis, methylation groups were re-assigned based on the 3-group methylation classifier. The boxes represent the interquartile range and whiskers depict the minimum and maximum of the distribution not considering outliers. Two-sided unpaired Welch’s t-test was used to test for expression differences between HM or IM vs. LM subgroups.

**SUPPLEMENTARY Figure 6 Aberrant DNA methylation patterns are associated with signaling pathway activation and over-expression of DNMTs**

**a** Heatmap depicting hierarchical clustering results of log2-transformed normalized gene expression of the 1000 most variably expressed genes in JMML (n=15, Euclidean distance metric, complete linkage). Relapse events (red) are annotated on top of the heatmap and methylation cluster assignment based on consensus clustering (k=2 [HM, LM] & k=3 [HM, IM, LM]) are annotated at the bottom of the heatmap.

**b** In order to visualize the global correlation of gene expression with DNA methylation, normalized log2-transformed gene expression values were binned from low expression (bin1) to high expression (bin10). For each expression bin, DNA methylation levels [%] are plotted as boxplots for all nvCpGs located in promoter regions. The boxes represent the interquartile range and whiskers depict the minimum and maximum of the distribution not considering outliers.

**c** Waterfall plot depicting the gene-wise Pearson correlation for DNA methylation levels with gene expression of the 1000 most variable promoter jmmlCpGs.

**d** Results from the gene set enrichment analysis (GSEA) using the ranked gene list from the analysis in panel **c** (the list was ranked according to Pearson correlation coefficient). and testing all MSigDB gene sets for enrichment. Depicted is the single significant enrichment result.

**
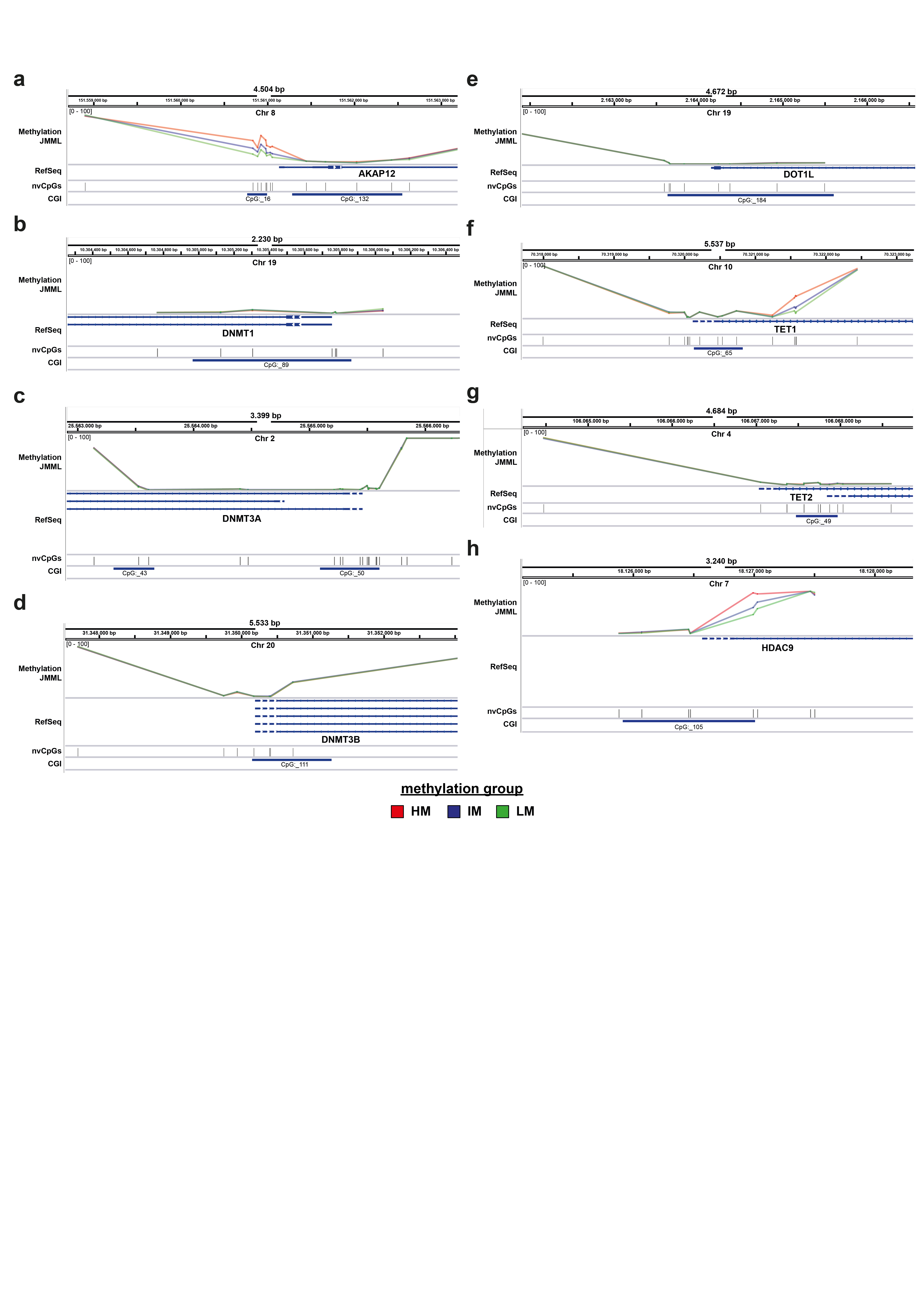
**

**SUPPLEMENTARY Figure 7 Aberrant DNA methylation patterns are associated with signaling pathway activation and over-expression of DNMTs**

**a-h** Browser screenshots depicting DNA methylation in promoter regions of candidate genes from **Figure 5f, g** and from **Supplementary Figure 5f**. Depicted are unsmoothed average DNA methylation levels for nvCpGs from the Illumina 450k bead chip arrays for samples from the validation cohort stratified for methylation group assignment (HM: red, IM: blue, LM: green). All browser screenshots also contain tracks depicting RefSeq genes, the position of individual nvCpGs and the position of CpG islands (CGI).

Supplementary Table 1: Clinical characteristics of the discovery cohort (n=20)

| **Spleen_ID** | **Age [years]** | **Sex** | **HbF [%]** | **HbF elevated for age** | **Karyotype** | **Leuko [x10^9/l]** | **Thrombo [x10^9/l]** | **BM Blasts [%]** | **PB Monocytes [x10^9/L]** | **Mutation Group** |
| --- | --- | --- | --- | --- | --- | --- | --- | --- | --- | --- |
| JMML_1 | 0.25 | m | n.a. | n.a. | normal | 31.0 | 78.0 | 1 | 4.7 | Noonan Syndrome * |
| JMML_2 | 0.28 | f | 48.6 | elevated | normal | 103.0 | 160.0 | 9 | 17.5 | *NF1* |
| JMML_3 | 0.45 | m | 5.9 | normal | normal | 34.1 | 93.0 | 3 | 12.3 | all neg |
| JMML_4 | 0.52 | f | 9.4 | elevated | 45,XX,-7 | 15.6 | n.a. | 1 | 7.0 | *KRAS* |
| JMML_5 | 0.53 | f | 1.4 | normal | normal | 42.0 | 530.0 | 3.5 | 2.5 | all neg |
| JMML_6 | 0.69 | m | 32.4 | elevated | normal | 57.6 | 75.0 | 4 | 5.8 | *KRAS* |
| JMML_7 | 1.14 | m | n.a. | n.a. | normal | 23.5 | 126.0 | 14 | 4.2 | *PTPN11* |
| JMML_8 | 1.93 | f | 10.3 | elevated | normal | 54.0 | 137.0 | 3 | 4.3 | *PTPN11* |
| JMML_9 | 1.93 | m | 13.9 | elevated | 45,XY,-7 | 10.6 | 93.0 | 3 | 5.0 | *KRAS* |
| JMML_10 | 2.18 | m | 21.8 | elevated | normal | 57.0 | 21.0 | 5 | 6.8 | *PTPN11* |
| JMML_11 | 2.33 | f | n.a. | n.a. | normal | 17.4 | 40.0 | 4 | 3.5 | *KRAS* |
| JMML_12 | 2.41 | m | n.a. | n.a. | normal | 13.6 | 60.0 | 0 | 1.8 | *KRAS* |
| JMML_13 | 2.48 | f | 36.3 | elevated | 45,XX,-7 | 13.2 | 74.0 | 3 | 2.9 | *PTPN11* |
| JMML_14 | 2.78 | m | n.a. | n.a. | 45,XY,-7 | 22.9 | 363.0 | 8 | 3.7 | all neg |
| JMML_15 | 3.74 | m | 59.5 | elevated | normal | n.a. | 103.0 | 13 | n.a. | *NF1* |
| JMML_16 | 5.04 | m | 9.2 | elevated | normal | 14.1 | 10.0 | 12 | 2.8 | *PTPN11* |
| JMML_17 | 5.36 | m | n.a. | n.a. | 45,XY,-21,inc | 33.6 | 21.0 | 1 | 5.0 | *KRAS* |
| JMML_18 | 5.46 | m | 5.9 | elevated | normal | 26.0 | 139.0 | 0 | 7.5 | *NF1* |
| JMML_19 | 6.08 | m | 70 | elevated | normal | 13.0 | 40.0 | 6 | 2.5 | *NRAS* |
| JMML_20 | 5.10 | f | n.a. | n.a. | n.a. | n.a. | n.a. | n.a. | n.a. | n.a. |

* clinical features & mutation

**Supplementary Table 2:** Summary of clinical characteristics of the validation cohort (n=147)

|  |  | **Total** | **HM-group** | **IM-group** | **LM-group** | **p** |
| --- | --- | --- | --- | --- | --- | --- |
|  | ***n*** | 147 | 40 | 45 | 62 |  |
| **Age at diagnosis [years]** | ***mean (range)*** | 1.4 (0.1-12.3) | 3.1 (1.0-12.3) | 1.4 (0.1-6.0) | 0.4 (0.1-3.6) | *<0.01* |
|  | ***< 2 yrs.*** | 95 (65%) | 5 (13%) | 32 (71%) | 58 (94%) | *<0.01* |
|  | ***>= 2 yrs.*** | 52 (35%) | *35 (88%)* | 13 (29%) | 4 (6%) |  |
| **Sex** | ***male*** | 103 (70%) | 30 (75%) | 33 (73%) | 40 (65%) | n.s. |
|  | ***female*** | 44 (30%) | 10 (25%) | 12 (27%) | 22 (36%) |  |
| **DNA source** | ***PB*** | 34 (23%) | 11 (28%) | 11 (24%) | 12 (19%) | n.s. |
|  | ***BM*** | 113 (77%) | 29 (73%) | 34 (76%) | 50 (81%) |  |
| **Leukocytes [10^9^/l]** | ***mean (range)*** | 32 (4-217) | 33 (4-217) | 30 (5-104) | 33 (8-162) | n.s. |
|  | ***< 12*** | 16 (11%) | 6 (15%) | 6 (13%) | 4 (7%) | n.s. |
|  | ***>= 12*** | 130 (89%) | 33 (85%) | 39 (87%) | 58 (94%) |  |
|  | ***missing*** | 1 | 1 | 0 | 0 |  |
| **Platelets [10^9^/l]** | ***mean (range)*** | 79 (5-548) | *38 (5-234)* | 99 (12-442) | 110 (9-548) | *<0.01* |
|  | ***< 70*** | 62 (47%) | *28 (78%)* | 18 (43%) | 16 (29%) | *<0.01* |
|  | ***>= 70*** | 71 (53%) | 8 (22%) | 24 (57%) | 39 (71%) |  |
|  | ***missing*** | 14 | 4 | 3 | 7 |  |
| **Hemoglobin [g/dl]** | ***mean (range)*** | 9.3 (3.4-101.0) | 9.4 (3.4-101.0) | 9.2 (3.4-13.5) | 9.2 (6.2-21.3) | n.s. |
|  | ***<10*** | 85 (63%) | 23 (68%) | 28 (67%) | 34 (59%) | n.s. |
|  | ***>=10*** | 49 (37%) | 11 (32%) | 14 (33%) | 24 (41%) |  |
|  | ***missing*** | 13 | 6 | 3 | 4 |  |
| **Myeloblasts (BM) [%]** | ***mean (range)*** | 4 (0-28) | 5 (1-28) | 5 (0-18) | 3 (0-20) | n.s. |
|  | ***<5%*** | 74 (53%) | 18 (45%) | 21 (48%) | 35 (63%) | n.s. |
|  | ***>=5%*** | 66 (47%) | 22 (55%) | 23 (52%) | 21 (38%) |  |
|  | ***Missing*** | 7 | 0 | 1 | 6 |  |
| **Monocytes (PB) [%]** | ***mean (range)*** | 19 (0-55) | 15 (5-31) | 26 (5-55) | 20 (0-38) | *<0.01* |
|  | ***<10%*** | 20 (14%) | 10 (25%) | 5 (11%) | 5 (8%) | *<0.01* |
|  | ***10-19%*** | 55 (37%) | 19 (48%) | 12 (27%) | 24 (39%) |  |
|  | ***>=20%*** | 72 (49%) | 11 (28%) | *28 (62%)* | 33 (53%) |  |
| **Spleen size at diagnosis** | ***mean (range)*** | 4 (0-14) | 5 (0-11) | 4 (0-14) | 4 (0-13) | n.s. |
| **[cm below the costal margin]** | ***<5 cm*** | 71 (55%) | 18 (50%) | 24 (55%) | 29 (59%) | n.s. |
|  | ***>=5 cm*** | 58 (45%) | 18 (50%) | 20 (46%) | 20 (41%) |  |
|  | ***missing*** | 18 | 4 | 1 | 13 |  |
| **Hemoglobin F** | ***normal*** | 43 (41%) | 0 (0%) | 13 (41%) | 30 (71%) | *<0.01* |
| **(age-adjusted)** | ***elevated*** | 63 (59%) | *32 (100%)* | 19 (59%) | 12 (29%) |  |
|  | ***missing*** | 41 | 8 | 13 | 20 |  |
| **Karyotype** | ***normal*** | 93 (72%) | 28 (76%) | 20 (47%) | 45 (90%) | *<0.01* |
|  | ***aberrant*** | 37 (29%) | 9 (24%) | *23 (54%)* | 5 (10%) |  |
|  | ***missing*** | 17 | 3 | 2 | 12 |  |
| **Mutation** | ***NF1*** | 14 (11%) | 5 (14%) | 7 (16%) | 2 (5%) | *<0.01* |
|  | ***PTPN11 som*** | 48 (39%) | *26 (70%)* | 16 (37%) | *6 (14%)* |  |
|  | ***KRAS som*** | 20 (16%) | 1 (3%) | *13 (30%)* | 6 (14%) |  |
|  | ***NRAS som*** | 19 (15%) | 3 (8%) | 2 (5%) | *14 (32%)* |  |
|  | ***CBL*** | 13 (11%) | 0 (0%) | *0 (0%)* | *13 (30%)* |  |
|  | ***No mutation*** | 10 (8%) | 2 (5%) | 5 (12%) | 3 (7%) |  |
|  | ***Noonan*** | 18 | 0 | 0 | *18* |  |
|  | ***incomplete*** | 5 | 3 | 2 | 0 |  |

**Supplementary Table 3:** Summary of treatment and outcome of non-syndromic JMML patients from the validation cohort (n=111)

|  |  | **Total** | **HM** | **IM** | **LM** | **p** |
| --- | --- | --- | --- | --- | --- | --- |
|  |  |  |  |  |  |  |
|  | ***n*** | 111 | 37 | 43 | 31 |  |
|  |  |  |  |  |  |  |
| **Survival** | ***alive*** | 82 (74%) | 23 (62%) | 33 (77%) | 26 (84%) | n.s. |
|  | ***dead*** | 29 (26%) | 14 (38%) | 10 (23%) | 5 (16%) |  |
|  |  |  |  |  |  |  |
| **Follow-up time from diagnosis [years]** | ***median (range)*** | 6.4 (0.0-15.9) | 7.3 (0.0-13.7) | 6.8 (0.0-14.8) | 5.5 (0.0-15.9) | n.s. |
|  |  |  |  |  |  |  |
| **HSCT performed** | ***No HSCT*** | 19 (17%) | 7 (19%) | 4 (9%) | 8 (26%) | n.s. |
|  | ***HSCT*** | 92 (83%) | 30 (81%) | 39 (91%) | 23 (74%) |  |
|  |  |  |  |  |  |  |
| **Conditioning** | ***BuCyMel*** | 79 (86%) | 25 (83%) | 34 (87%) | 20 (87%) | n.s. |
|  | ***Bu and/or Cy, no Mel*** | 11 (12%) | 5 (17%) | 4 (10%) | 2 (9%) |  |
|  | ***other*** | 2 (2%) | 0 | 1 (3%) | 1 (4%) |  |
|  |  |  |  |  |  |  |
| **Alive after HSCT** | ***yes*** | 70 (76%) | 21 (70%) | 30 (77%) | 19 (83%) | n.s. |
|  | ***no*** | 22 (24%) | 9 (30%) | 9 (23%) | 4 (17%) |  |
|  | ***no HSCT*** | 19 | 7 | 4 | 8 |  |
|  |  |  |  |  |  |  |
| **Event after HSCT** | ***no event**** | 56 (61%) | 13 (43%) | 26 (67%) | 17 (74%) | *<0.01* |
|  | ***TRM*** | 13 (14%) | 2 (7%) | 7 (18%) | 4 (17%) |  |
|  | ***relapse*** | 23 (25%) | *15 (50%)* | 6 (15%) | 2 (9%) |  |
|  | ***no HSCT*** | 19 | 7 | 4 | 8 |  |

****1 pGF alive***
