# Supplementary material for: RAS-pathway mutation patterns define epigenetic subclasses in juvenile myelomonocytic leukemia
Source: Nat Commun. 2017 Dec 19;8:2126. doi: 10.1038/s41467-017-02177-w (PMC5736667; doi:10.1038/s41467-017-02177-w)
Supplement: Supplementary file 3 — Description of Additional Supplementary Files [file 41467_2017_2177_MOESM3_ESM.docx]

**Description of Additional Supplementary Files**

File Name: Supplementary Data 1

Description: Annotated list of all nvCpGs

File Name: Supplementary Data 2

Description: Annotated list of all JMML-specific DMPs

File Name: Supplementary Data 3

Description: Patient’s characteristics for the validation cohort
